# Association of anxiety with intracortical inhibition and descending pain modulation in chronic myofascial pain syndrome

**DOI:** 10.1186/1471-2202-15-42

**Published:** 2014-03-19

**Authors:** Liliane Pinto Vidor, Iraci LS Torres, Liciane Fernandes Medeiros, Jairo Alberto Dussán-Sarria, Letizzia Dall’Agnol, Alicia Deitos, Aline Brietzke, Gabriela Laste, Joanna R Rozisky, Felipe Fregni, Wolnei Caumo

**Affiliations:** 1 Post-Graduate Program in Medical Sciences, School of Medicine, Universidade Federal do Rio Grande do Sul, Porto Alegre, Brazil; 2 Post-Graduate Program in Biologic Sciences: Physiology, Institute of Basic Health Sciences, Universidade Federal do Rio Grande do Sul, Porto Alegre, Brazil; 3 Pharmacology Department, Institute of Basic Health Sciences, Universidade Federal do Rio Grande do Sul, Endereço: Rua Sarmento Leite, 500/202 – CEP, Porto Alegre 90050-170, Brazil; 4 Laboratory of Pain and Neuromodulation, Hospital de Clínicas de Porto Alegre (HCPA), Porto Alegre, Brazil; 5 Department of Physical Medicine and Rehabilitation, Harvard Medical School, Boston, Massachusetts, United States of America; 6 Pain and Palliative Care Service at Hospital de Clínicas de Porto Alegre (HCPA), Universidade Federal do Rio Grande do Sul (UFRGS), Rua Ramiro Barcelos, 2350, Bairro Santa Cecilia, Porto Alegre, Rio Grande do Sul CEP 90035-903, Brazil

**Keywords:** Transcranial magnetic stimulation, Chronic pain, Noninvasive brain stimulation, Neuromodulation, Anxiety, Myofascial pain syndrome

## Abstract

**Background:**

This study aimed to answer three questions related to chronic myofascial pain syndrome (MPS): 1) Is the motor cortex excitability, as assessed by transcranial magnetic stimulation parameters (TMS), related to state-trait anxiety? 2) Does anxiety modulate corticospinal excitability changes after evoked pain by Quantitative Sensory Testing (QST)? 3) Does the state-trait anxiety predict the response to pain evoked by QST if simultaneously receiving a heterotopic stimulus [Conditional Pain Modulation (CPM)]? We included females with chronic MPS (n = 47) and healthy controls (n = 11), aged 19 to 65 years. Motor cortex excitability was assessed by TMS, and anxiety was assessed based on the State-Trait Anxiety Inventory. The disability related to pain (DRP) was assessed by the Profile of Chronic Pain scale for the Brazilian population (B:PCP:S), and the psychophysical pain measurements were measured by the QST and CPM.

**Results:**

In patients, trait-anxiety was positively correlated to intracortical facilitation (ICF) at baseline and after QST evoked pain (β = 0.05 and β = 0.04, respectively) and negatively correlated to the cortical silent period (CSP) (β = -1.17 and β = -1.23, respectively) (P <0.05 for all comparisons). After QST evoked pain, the DRP was positively correlated to ICF (β = 0.02) (P < 0.05). Pain scores during CPM were positively correlated with trait-anxiety when it was concurrently with high DRP (β = 0.39; P = 0.02). Controls’ cortical excitability remained unchanged after QST.

**Conclusions:**

These findings suggest that, in chronic MPS, the imbalance between excitatory and inhibitory descending systems of the corticospinal tract is associated with higher trait-anxiety concurrent with higher DRP.

## Background

Pain is not simply determined by the intensity of the nociceptive stimulus but also by orchestrated mechanisms that work together, including psychological factors [1]. As one of these psychological factors, anxiety involves both physiological and psychological aspects that affect the way sensory interpretation occurs [2]. Anxiety is considered adaptive because a threatening situation induces body changes that increase the state of arousal. Anxiety can be presented as a state-anxiety (i.e., referred to acute situation-driven episodes that fluctuate over time) or as a trait-anxiety (i.e., a lifelong pattern, in the form of a personality feature) [3]. Maladaptive anxiety can take over and have negative effects on people’s lives [4].

These descriptions are consistent with the State-Trait Anxiety theory, which predicts that individuals with high trait-anxiety are generally hypersensitive to stimuli and are psychologically more reactive [3,4]. Anxious patients present signs of restlessness, sympathetic overactivity, and resistance to sedation [5]. Thus, similarly to pain mechanisms, it is conceivable that anxiety is associated with alterations in brain excitability [5]. Previous reports observed that the motor-evoked potentials (MEP) amplitude was correlated with neuroticism in subjects with anxiety-related personality traits [6] and that short intracortical inhibition (SICI) was decreased in those with obsessive-compulsive disorder (OCD) when compared to subjects who were screened as psychiatrically normal [7].

In the clinical setting, subjects who frequently experience stress and anxiety have higher predisposition to develop trigger points, which can lead to myofascial pain syndrome (MPS) [8]. According to epidemiologic studies, the myofascial trigger points (MTrPs) might be a source of nociceptive inputs in 30% to 85% of the patients seeking pain relief [9,10]. A common sign of MPS is the referred pain to distant somatic structures with concomitant modifications of superficial and deep sensitivity in the painful areas [11-13]. It has been theorized that there are two main mechanisms for pain hypersensitivity in muscle pain syndromes: a “bottom-up mechanism” in which the process starts and is maintained by changes in deep peripheral tissues, and the “top-down mechanism” which claims that the origin lies in the stress- and pain- regulating systems in the brain [14]. Both mechanisms would induce changes in the excitability of nociceptive neurons in the dorsal horn, descending and ascending corticospinal tracts and cortical structures, leading to hypersensitization. To study the inhibitory mechanisms that modulate pain processing at the spinal cord, a “conditioned pain modulation (CPM) stimulus” [15] method has been used in which heterotopic noxious stimuli applied to a remote area of the body was shown to attenuate the activity of pain-signaling neurons in the spinal dorsal horn [16-18].

To improve the understanding of the central mechanisms related to anxiety and pain, we assessed cortical excitability parameters using single and paired pulse transcranial magnetic stimulation (TMS). We hypothesize that corticospinal excitability is modulated by anxiety favoring loss of descendent inhibitory influx. The study presented here aimed to answer three questions: 1) Is the motor cortex excitability, as assessed by transcranial magnetic stimulation parameters (TMS), related to state-trait anxiety? 2) Does anxiety modulate corticospinal excitability changes after evoked pain by Quantitative Sensory Testing (QST)? 3) Does the state-trait anxiety predict the response to pain evoked by QST if simultaneously receiving a heterotopic stimulus [Conditional Pain Modulation (CPM)]?

## Methods

### Study design

The study protocol was reviewed and approved by the ethics committee at the Hospital de Clínicas de Porto Alegre (HCPA) in Porto Alegre, Brazil (Protocol No. 10-0196), where the study was carried out. All the procedures were conducted in accordance with the Declaration of Helsinki. All the subjects gave their written informed consent prior to participation.

### Study sample

Patients were recruited from the general population using public postings in different health care units and referrals from physicians in the Chronic Pain Service at HCPA. Inclusion criteria included right-handed female subjects aged 19 to 65 years, with a confirmed diagnosis of MPS in the upper body segment for at least three months prior to enrollment, and limited routine activities due to MPS. The limitation in activities was evaluated by asking subjects specifically for the presence (i.e., yes or no) of symptoms that interfere with work, personal relationships, enjoyment of activities, responsibilities at home, personal goals, and clear thinking (i.e., problem solving, concentrating, and/or remembering) during the past three months. Only subjects with the presence of interference in one or more of the activities assessed were included. The diagnosis of MPS was confirmed by a second independent examiner with more than 10 years of clinical experience related to chronic pain. MPS was defined by regional pain, normal neurological examination, decreased range of motion, stiffness in the target muscles, presence of trigger points, taut bands, tender points, palpable nodules, and pain characterized as *dull*, *hollow*, or *deep* that exacerbates during stress. To distinguish neuropathic pain from ongoing nociception, the Neuropathic Pain Diagnostic Questionnaire (DN4) was applied to all patients. Only those with a neuropathic component (score greater than or equal to 4) were included [19]. The exclusion criteria included the presence of any other pain disorder, such as rheumatoid arthritis, radiculopathy, and fibromyalgia; previous surgery on the affected areas; and the regular use of anti-inflammatory steroids (because it may interfere with TMS results). In addition, patients with contraindications for TMS were also excluded [20].

Healthy controls were recruited from the general population using public postings as well. They were asked to complete screening questionnaires, and were excluded if they were experiencing any painful condition (either acute or chronic); used analgesics or corticosteroids; had any rheumatologic, psychiatric, or neurological disorder; had abused of alcohol or psychotropic substances during the six months previous to the screening; or if they were using medications with known effects on the central nervous system (CNS).

Considering type I and II errors of 0.05 and 0.20, respectively, and anticipating an effect size (*f*^2^) of 0.25 for multiple regression analysis allowing for two predictors (Post-hoc Statistical Power Calculator for Hierarchical Multiple Regression: http://www.danielsoper.com/statcalc3/calc.aspx?id=17[21], the sample size of 42 patients was estimated. A sample of 47 patients was determined as to account for unexpected factors that would decrease study power such as increased variability of our sample or missing data. In fact, a sample of 47 patients would detect an effect size for correlations of 0.25 with a power of 84% at a 0.05 alpha level.

### Assessments

#### Demographic characteristics, depressive symptoms, pain-related catastrophic thinking and anxiety

**Figure 1:**
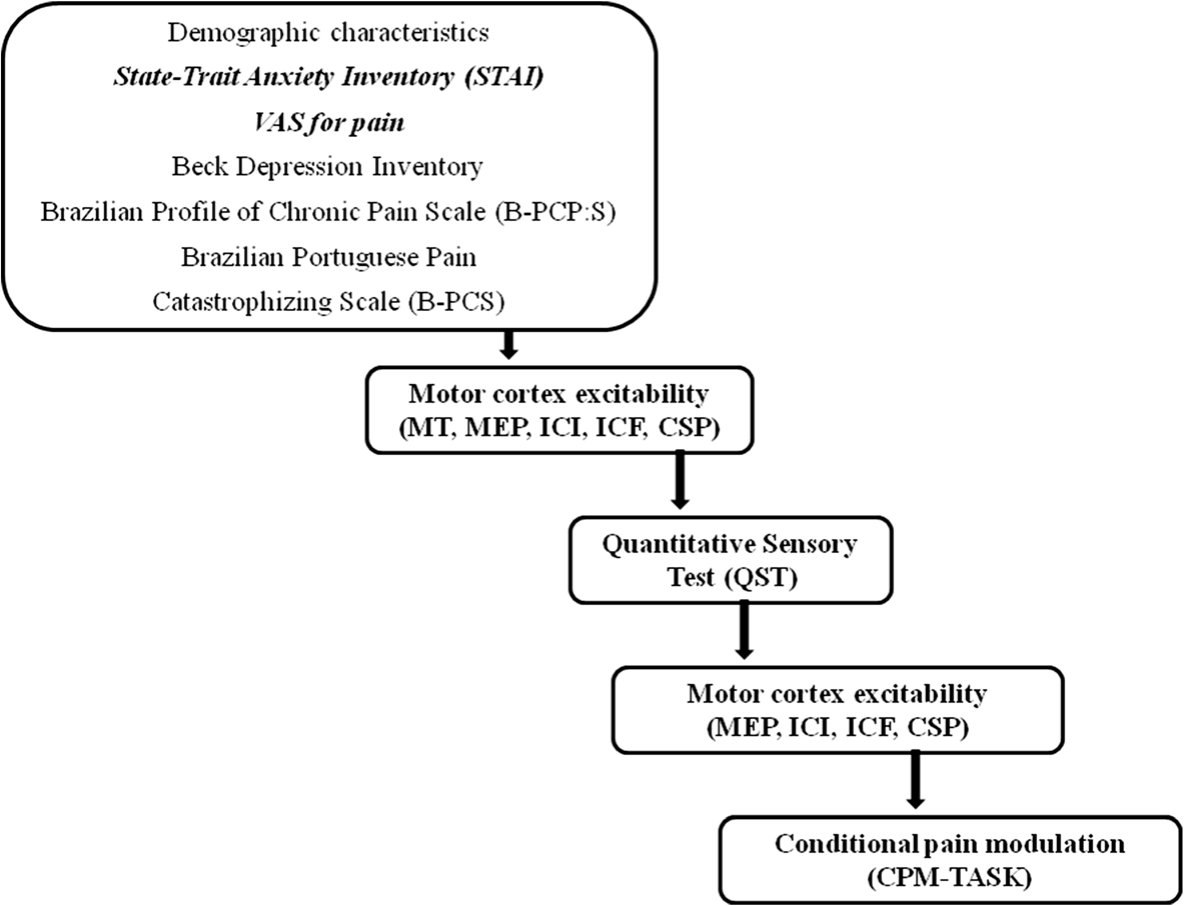
Flow of sequence of study steps.

Demographic information was gathered using a standardized questionnaire. All the psychological instruments used in this study have been validated for the Brazilian population and were applied by trained evaluators. The instruments have been validated by our group [22,23]. Pain-related catastrophic thinking was assessed using the Brazilian Portuguese Catastrophizing Scale (B-PCS) [22]. The disability related to pain (DRP) in terms of severity, interference with daily activities, and emotional burden was evaluated using the Profile of Chronic Pain: Screen for a Brazilian Population (B-PCP:S) [23]. An accepted criterion to define disability related to pain is a chronic or recurrent pain or discomfort causing restriction [23], thus we assumed that higher scores on the B-PCP:S indicated higher disability or functionality at work, at home, during social situations and/or a higher emotional burden. The Beck Depression Inventory was employed to assess depressive symptoms [24]. The sequence of assessments is presented in Figure 1. Healthy controls underwent the same sequence of assessments, excepting the questionnaires regarding pain and depression; the conditioned pain modulation test and the TMS paired pulse assessments.

a) Anxiety was measured with the State-Trait Anxiety Inventory (STAI), adapted to Brazilian Portuguese [25]. State-anxiety (a situation-driven transient anxiety) and trait-anxiety (stable personality disposition reflecting general level of fearfulness) were evaluated. When answering the S-Anxiety Scale, subjects chose the number that best describes the intensity of their feelings in a four-point Likert scale, as follows: (1) not at all, (2) somewhat, (3) moderately, (4) and very much so, for 13 different items. When answering the T-Anxiety Scale, subjects rate the frequency of their feelings through 12 different items using the following four-point Likert scale: (1) almost never, (2) sometimes, (3) often, (4) almost always. Because the Rash analysis pooled two optios for some of the items of the STAI, some of the questions in the reduced version are rated as sometimes (2)/often (3). Item scores are added to obtain subtest total scores, taking into account that 10 out of 25 responses should be reversed for the anxiety-absent questions. The S-Anxiety scale score ranges from 13 to 52, and the T-Anxiety scale score ranges from 12 to 36. Higher scores denote higher levels of anxiety.

b) The global intensity of pain was measured with the Visual Analogue Scale (VAS). The scores for pain range from 0 (no pain) to 10 (worst pain possible).

c) Quantitative Sensory Testing (QST) was used to assess heat pain thresholds using the method of limits with a computer Peltier-based device thermode (30 × 30 mm) [26] that was attached to the skin on the ventral aspect of the mid-forearm. The baseline temperature was set at 32°C and was increased at a rate of 1°C/s to a maximum of 52°C. The patient had to report when she began to feel a warm sensation and when it became painful, with the latter representing the heat pain threshold (HPT). Three assessments were performed with an inter-stimuli interval of 40 seconds [26]. Each subject’s HPT was defined as the mean painful temperature of the three assessments. The position of the thermode was slightly altered between trials (although it remained on the left ventral forearm) to avoid either sensitization or response suppression of the cutaneous heat nociceptors.

d) QST during cold water immersion (CPM-TASK): The temperature at which subjects felt 6/10 on the numerical pain scale (NPS) ranging from 0 (no pain) to 10 (the worst pain imaginable) was assessed. By measuring QST during cold-water immersion, we evaluated the degree to which pain perception is modulated following the presentation of an initial heterotopic noxious stimulus (Conditional Pain Modulation - CPM). Subjects immersed their non-dominant hands into cold water (zero to one degree Celsius) for one minute. The QST procedure was administered after 30 seconds of the cold-water immersion. During this test, subjects were asked to rate the pain of the stimulated hand using the same NPS. The temperature was held constant during the experiment for each subject. Differences (presented in percentage) between the average pain rating before and after cold water immersion was defined as the CPM.

e) Analgesic use was defined as the self-reported average of analgesics used per week during the last three months. For data analysis, analgesic use was included as a dichotomous variable in which the use of analgesics less than four days per week was coded as zero (reference value) and their use on more than four days per week was coded as one. This strategy was chosen because subjects with chronic pain typically use rescue analgesics irregularly and their frequency of use changed each week according to their pain level.

### Cortical excitability

Transcranial Magnetic Stimulation (TMS) of the left motor cortex (M1) was performed using a MagPro X100 stimulator (MagVenture Company, Lucernemarken, Denmark) through a figure-of-eight coil (MagVenture Company). It was assessed at baseline, at QST assessment and after the CPM intervention. Ag-AgCl electrodes were placed over the first dorsal interosseus (FDI) muscle belly and its corresponding tendon on the distal phalanx of the index finger. Responses to stimuli were recorded from the FDI muscle of the right hand by surface electromyography (EMG).

The subjects were seated in a comfortable chair and were informed about the TMS procedure, including all sensations that may be felt. The amplitudes of single and paired pulse TMS and the latency of cortical silent period were measured during the experiment and recorded on a excel spreadsheet. Data were analyzed offline on a personal computer.

The coil was placed tangentially to the scalp over the left M1, with an angle of 45° to the sagittal line to identify the motor “hot spot”, which was defined as the coil position over the left M1 in which the lowest Motor Threshold (MT) intensity was required to elicit an acceptable response in at least 50% of the evoked potentials of the resting FDI [27,28]. This site was marked with a soft-tipped pen to ensure a constant placement of the coil throughout the TMS assessments. First, the motor threshold (MT) was determined, which was defined as the lowest stimulus intensity sufficient to elicit a response of at least 5 of 10 evoked potentials (at least 50% of successive trials) with minimum amplitude of 50 μV peak-to-peak in the resting FDI [27,28]. Then, single-pulse measures including the Motor Evoked Potential (MEP) and Cortical Silent Period (CSP) were recorded at an intensity of 130% of the MT. The MEP value was the one that elicited the evoked potential with 1 mV peak-to-peak amplitude. The means of ten consecutive trials were recorded. For the CSP the patients were instructed to perform isometric voluntary contraction with approximately 10% of maximal contraction of the FDI. The transient silence in isometric voluntary EMG activity was elicited in the tonically contracting FDI muscle with about 10% of the maximal voluntary contraction; the CSP was preceded by the MEP [29]. Also, ten consecutive trials were recorded. Paired-pulse measures including the Short Intracortical Inhibition (SICI) with interstimuli interval of 2 ms and Intracortical Facilitation (ICF) with interstimuli interval of 12 ms. The first sub-threshold stimulus was set at 80% of the individual MT, and the second supra-threshold stimulus was set at 130% of the MT. The intensity of supra-threshold test stimuli was adjusted to elicit test stimuli with peak-to-peak amplitude of about 1 mV. The reduction of the test MEP elicited by TMS is considered to reflect inhibition at the primary motor cortex [30], and increments of the test MEP elicited by TMS are considered to reflect facilitation at the primary motor cortex [31]. Thirty recordings (ten for each SICI, ICF, and test stimuli) were made in random order having an interval of approximately 8 seconds between each pulse. Paired-pulse measures were analyzed calculating their individual index (Mean SICI/Mean of test stimuli; Mean ICF/Mean of test stimulus) [30,32]. The same MT value was used to elicit MEP, CSP, SICI and ICF at baseline, at QST assessments and after the intervention.

Three QST assessments were performed with an inter-stimuli interval of 40 seconds and the MEP was assessed shortly after these measurements. The same researcher performed the TMS assessments for each patient intending to reduce potential between-evaluators variability.

### Statistical analyses

Descriptive statistics were used to summarize the main features of the sample. Continuous data were evaluated for normality using Skewness/Kurtosis tests. After verifying the corresponding assumptions, linear regression analysis with forward selection controlling for collinearity [33] was used to identify potential confounding factors in the association between the main independent variables of interest, state-trait anxiety, and the dependent variables MT, MEP, ICF, SICI and CSP. The covariates included in the models were age, depressive symptoms, and catastrophizing thinking due to chronic pain. Only covariates retained in each one these models (Table 1) were included in a final multivariate linear regression model with cortical excitability parameters as dependent variables (i.e., MT, MEP, CSP, SICI and ICF) and age, pain on the B:PCP:S and trait-anxiety as independent variables. The B:PCS was excluded after collinearity with the B:PCP:S was identified.

**Table 1:** Linear regression of the relationship between cortical excitability parameters and potential confounding factors (n = 47)

**Parameter**	**SEM**	**β**^**a**^	**t**	***P***
**Motor Threshold**				
Age (years)	0.09	0.31	2.15	0.03
**Short Intracortical inhibition** (ratio: SICI/test stimulus)				
Brazilian Portuguese Catastrophizing Scale (B-PCS)	−0.005	−0.44	−3.24	0.002
**Intracortical facilitation**				
(ratio: ICF/test stimulus)				
Trait-anxiety	0.011	0.56	4.48	0.001
**Cortical silent period**				
Trait-anxiety	0.39	−0.41	−2.95	0.005

^*a*^*Value adjusted by multiple regression analysis with a stepwise forward method controlling for collinearity. The covariates included in each model were: state-trait anxiety, pain-related catastrophic thinking, depressive symptoms and age.*

The change in MEP was calculated as the standardized mean difference (SDM) expressed as a percentage (%) following the formula [(MEP before QST evoked pain minus MEP after)/(Standard deviation of the MEP before QST evoked pain)] multiplied by 100. The percentage of the MEP change was defined as a dependent variable. Thus, a multiple regression analysis was run to assess the association between the percentage of MEP change and trait-anxiety, adjusting for B-PCP:S and age.

The changes in MEP before and after QST evoked were compared using a paired *t*-test. Additionally, the relationships between CPM, trait-anxiety, DRP and number of days analgesics used per week in the last three months (< 4 times/≥4 times) were assessed using multiple linear regression models. The data was analyzed using SPSS version 18.0 (SPSS, Chicago, IL).

## Results

We screened 62 potential participants with a diagnosis of MPS, and forty-seven of them were included in the study. The reasons for exclusion were not fulfilling diagnostic criteria for MPS, lack of disability as defined in the protocol, inability to demonstrate a neuropathic component as assessed by DN4 (Neuropathic Pain Diagnostic Questionnaire) or the presence of another diagnosis (fibromyalgia).

All subjects who enrolled also concluded the study and were included in all the analyses (Table 2). Motor cortical excitability parameters are presented in (Table 3).

**Table 2:** Sample characteristics

**Variables**	**Mean (SD) or percentage**	**Median (Q25, Q75)**
Age (years)	47.28 (11.51)	48 (39, 56)
Marital status (married/unmarried)	15/47 (yes: 31.91%)	---
Weight (kg)	65.69 (11.59)	65 (56.5, 75)
Height (m)	1.62 (0.17)	1.6 (1.56, 1.64)
Education (years)	13.19 (4.20)	13 (11, 16)
Trait-anxiety	23.89 (6.90)	22.5 (19, 29)
State-anxiety	28.22 (7.80)	26 (22, 34)
Beck depression inventory	15.02 (9.27)	13.5 (9, 19)
Brazilian Portuguese Catastrophizing Scale (B-PCS)	29.36 (12.59)	33 (20, 39)
Pain lasting longer than one year (yes/no)	40/48 (yes: 83.33%)	---
Total score on the Profile of Chronic Pain: Screen for Brazilian population (B-PCP:S)	59.70 (15.75)	62 (49, 71)
Score on B-PCP:S domains		
Intensity	24.6 (3.3)	25 (23, 27)
Interference in daily activity	21.98 (9.35)	24 (18, 30)
Emotional burden	13.11 (6.24)	13 (9, 18)
Number of days analgesics were used per week in the last three months (< 4 times/≥ 4 times)^a^	22/47 (< 4 times: 46.81%)	---
Smoking (yes/no)	26/47 (yes: 55.32%)	---
Alcohol consumption (yes/no)	26/47 (yes: 55.32%)	---
Presence of other chronic diseases before appearance of pain (yes/no)^b^	21/47 (yes: 44.68%)	---
Diagnosis of psychiatric disorders (yes/no)	18/47 (yes: 38.30%)	---
Active use of central nervous system medication (yes/no)^c^	4/47 (yes: 8.51%)	---

^*a*^*The same patient may have used more than one medication.*

^*b*^*Chronic diseases other than pain: hypertension (n = 12); ischemic heart disease (n = 1); heart attack (n = 1); diabetes mellitus (n = 5); thyroid diseases (n = 2); other chronic diseases listed (n = 0).*

^*c*^*Central nervous medication: tricyclic antidepressant (n = 2); topiromate (n = 1) tylex (n = 1).*

Data presented as Mean (SD) or median (interquartile) or proportion (n = 47).

**Table 3:** Measurements of motor cortex parameters using transcranial magnetic stimulation (n = 47)

**Cortical excitability measures**				
	**Before QST evoked pain**		**After QST evoked pain**	
	**Mean±SD**	**Median (Q25, Q75)**	**Mean±SD**	**Median (Q25, Q75)**
Motor Threshold (MT)	42.65 (7.39)	41 (37.5, 46)	----	-----
Motor evoked potential (mV)	1.74 (0.71)	1.53 (1.27, 2.20)	2.13 (0.93)	1.93 (1.58, 2.58)
Intracortical Facilitation (ratio: ICF/test stimulus)	1.16 (0.42)	1.11 (1, 1.23)	1.25 (0.59)	1.15 (0.92, 1.44)
Short Interval Intracortical Inhibition (ratio: SICI/test stimulus)	0.32 (0.14)	0.28 (0.22, 0.35)	0.34 (0.19)	0.29 (0.21, 0.43)
Cortical Silent Period (CSP)	65.42 (19.64)	66.25 (47.9, 79.31)	66.16 (20.16)	68.30 (50.59, 81.71)

*(Motor evoked potential: MEP); Interquartile interval (Q); Intra-cortical inhibition (ICI) expresses the relationship between the amplitude of wave and motor evoked potentials (relative amplitude, express in%), at inter-stimuli intervals (ISIs) of 2 ms with paired-pulse. The first is a sub-threshold stimulus [80*% *of the rest motor threshold (rMT)] followed by the second one which is a suprathreshold stimulus (130*% *rMT).****(B)****Cortical silent period (CSP) expressed in milliseconds (ms);****(C)****Motor-evoked potentials (MEP) expressed in mV, evoked by a stimulus of 130*% *the intensity of the rMT, and should have peak-to-peak MEP amplitude of at least 1 mV.*

**Figure 2:**
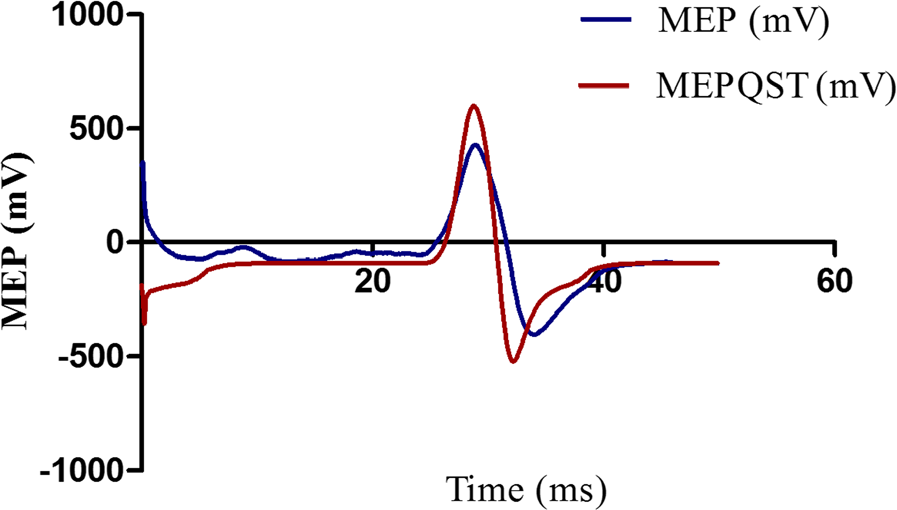
Example of 10 averaged transcranial magnetic stimulus-induced Motor Evoked Potentials at the First Dorsal Interosseus (FDI), before (MEP) and after (MEPQST) the Quantitative Sensory Testing (QST).

In Figure 2 are presented the MEP amplitude in mV, before and after QST of a representative patient. This data presented is an average of 10 trials before and after QST. Patients presented higher MEPs amplitudes after QST in relation to the amplitudes before.

**Figure 3:**
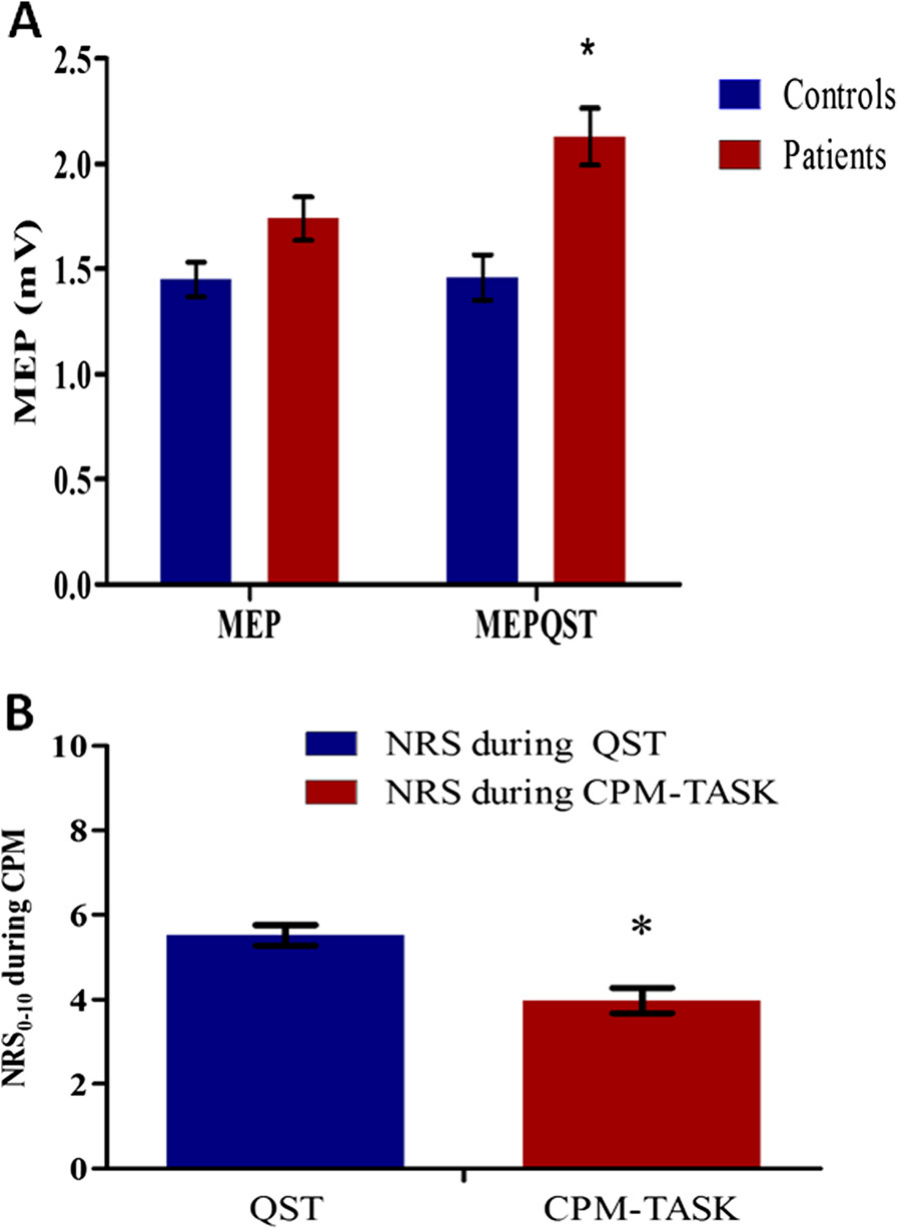
**Changes on cortical excitability and pain during the QST and CPM-TASK. (A)** Motor-evoked potentials (MEP) expressed in mV, evoked by a stimulus of 130% of the rMT, before and during the heat pain threshold induced by the Quantitative Sensory Test (QST) in patients and healthy controls. Bars represent the standard error of the mean (S.E.M.). Asterisks positioned above the bars indicate a significant difference (P < 0.05) between the MEP amplitude before and after QST. **(B)** Reduction in pain intensity during the Conditioned Pain Modulation (CPM). The pain on the numeric rating scale (NRS) from 0 to 10 is presented during the test stimulus (QST) and during the conditioning stimulus (cold-pressor task, CPM-TASK). Bars express the standard error of the mean (S.E.M.) (n = 47). Asterisk positioned above the bars indicate a significant difference (P < 0.05) between the NRS during the CPM.

Eleven healthy controls with mean (standard deviation) age of 26.27 (6.31) were recruited. Their mean trait-anxiety score was 17.18 (3.43), and state-anxiety score 21.55 (4.72). Their MEP remains unchanged before and after the QST (P > 0.05) (Figure 3).

### Relationship between motor cortex excitability and state-trait anxiety level

As described in the statistical analysis section, potential confounding factors in the relationship between the independent variables of major interest (state-trait anxiety) and the cortical excitability parameters [MT (resting motor threshold), MEP, ICF, ICI and CSP] were identified using linear regression model analysis (Table 1). When the outcome variable was the MEP, no other variables were retained in the model.

The multivariate linear regression model with the cortical excitability parameters obtained at baseline (before QST evoked pain) as dependent variables (MT, MEP, ICF, SICI, CSP) using age, trait-anxiety and pain on B:PCP:S as independent variables (Table 4). This analysis revealed a significant relationship between trait-anxiety and cortical excitability measurements (Wilks’ λ = 0.84, F (34) = 34.7, P < 0.0001). The power of this analysis was 0.84. Table 4B presents the results of the multivariate regression model between cortical excitability parameters (MEP, ICF, SICI, CSP) after QST evoked pain using age, trait anxiety and score on B:PCP:S as independent variables. In both conditions, at baseline (before QST evoked pain) and after QST evoked pain, the trait-anxiety was associated with motor cortex disinhibition, as indicated by a positive correlation with ICF and a negative correlation with CSP (Table 4). Also, the ICF was positively correlated with DRP after the QST evoked pain (Table 4). This analysis revealed a significant relationship between trait-anxiety and cortical excitability (Wilks’ λ = 0.80, F (4) = 38, P < 0.0001). The power of this analysis was 0.80.

**Table 4:** Multivariate linear regression analysis of the relationship between trait anxiety and motor cortex excitability before QST evoked pain (n = 47)

**Dependent variable**	**Type III sum of squares**	**df**	**Mean square error**	**F**	***P***	***Partial eta squared***
Motor threshold	256.75	4	64.19	1.12	0.36	0.10
Motor evoked potential	1.93	4	0.48	0.87	0.48	0.08
Intracortical facilitation	6.25	4	1.56	5.69	0.001*	0.37
Short intracortical inhibition	0.228	4	0.057	3.61	0.01*	0.28
Cortical silent period	5466.5	4	1366	5.67	0.001*	0.37
	**β**	**SEM**	**t**		***P***	***Partial eta squared***
**Motor threshold**						
Trait-anxiety	−0.06	0.17	−0.38		0.70	0.004
Age (years)	0.17	0.09	1.84		0.07	0.08
*B-PCP:S*	0.09	0.07	1.36		0.18	0.04
**Motor evoked potential**						
Trait-anxiety	−0.02	0.02	−0.78		0.44	0.02
Age (years)	−0.01	0.009	−1.37		0.17	0.04
*B-PCP:S*	−0.007	0.007	−1.05		0.29	0.03
**Intracortical Facilitation**^***c***^						
Trait-anxiety	0.05	0.012	4.06		0.00*	0.29
Age (years)	−0.008	0.007	−1.19		0.23	0.03
*B-PCP:S*	−0.004	0.005	−0.77		0.45	0.01
**Short intracortical inhibition**^***c***^						
Trait-anxiety	0.001	0.003	0.32		0.75	0.003
Age (years)	0.00	0.002	0.21		0.83	0.001
*B-PCP:S*	−0.002	0.001	−1.21		0.23	0.04
**Cortical silent period**						
Trait-anxiety	−1.17	0.39	−2.99		0.01*	0.18
Age (years)	-.013	0.22	−0.06		0.95	0.00
*B-PCP:S*	−0.163	0.16	−1.02		0.31	0.03
**Dependent variable**	**Type III sum of squares**	**df**	**Mean square error**	**F**	***P***	***Partial eta squared***
Motor evoked potential	10.48	3	3.49	4.93	0.13	0.12
Intracortical facilitation	4.27	3	1.42	5.08	0.004*	0.27
Short intracortical inhibition	0.04	3	0.012	0.31	0.81	0.02
Cortical silent period	2979.0	3	993.01	2.62	0.06	0.16
	**β**	**SEM**	**t**		***P***	***Partial Eta Squared***
**Motor evoked potential**						
Trait-anxiety	−0.04	0.02	−2.01		0.05	0.09
Age (years)	−0.006	0.01	−0.53		0.59	0.01
*B-PCP:S*	−0.04	0.08	−0.28		0.78	0.01
**Intracortical facilitation**^***c***^						
Trait-anxiety	0.04	0.01	3.09		0.01*	0.19
Age (years)	0.002	0.007	0.32		0.75	0.01
*B-PCP:S*	0.02	0.005	3.16		0.01*	0.19
**Short intracortical inhibition**^***c***^						
Trait-anxiety	0.003	0.005	0.57		0.57	0.008
Age (years)	0.00	0.003	−0.07		0.95	0.001
*B-PCP:S*	−0.002	0.002	−0.88		0.38	0.01
**Cortical silent period**						
Trait-anxiety	−1.23	0.45	−2.72		0.01*	0.15
Age (years)	−0.08	0.26	−0.31		0.75	0.002
*B-PCP:S*	0.05	0.18	0.25		0.80	0.001

^*b*^*Profile of Chronic Pain: Screen for a Brazilian population (B-PCP:S);*^*c*^(ratio: ICF/test stimulus; ratio: SICI/test stimulus). *P < 0.05

### Relationship between anxiety and the corticospinal modulatory system

The relationship between trait-anxiety and the change in MEP (pre to after QST evoked pain) was analyzed in the regression model using the latter as the dependent variable. The independent variables were age and pain as reported on the B-PCP:S (Screen for a Brazilian Population). The MEP amplitudes was significantly associated with trait-anxiety: F (48,1) = 5.86, β = 0.36; t = 2.42, P = 0.02. The independent variable retained in the model was the trait-anxiety. The positive correlation between trait-anxiety and the MEP indicates that higher trait-anxiety scores are associated with a greater change on the MEP amplitudes after QST evoked pain. The trait-anxiety explained 36% of the variance in the MEP amplitude induced by QST evoked pain.

The effect of the QST evoked pain on the corticospinal modulatory system was evaluated by comparing data before and after the QST evoked pain (Figure 2). There was an increase in MEP amplitudes after the QST evoked pain (P < 0.05), indicating the higher excitability of the motor cortex. The increased intracortical inhibition induced by CPM was demonstrated by a higher pain threshold during the CPM-TASK, as shown by lower pain scores on the NPS when compared to the NPS evoked by the QST alone (P < 0.001) (Figure 3).

### Effects of analgesics on the relationship between anxiety and the corticospinal modulatory system

**Figure 4:**
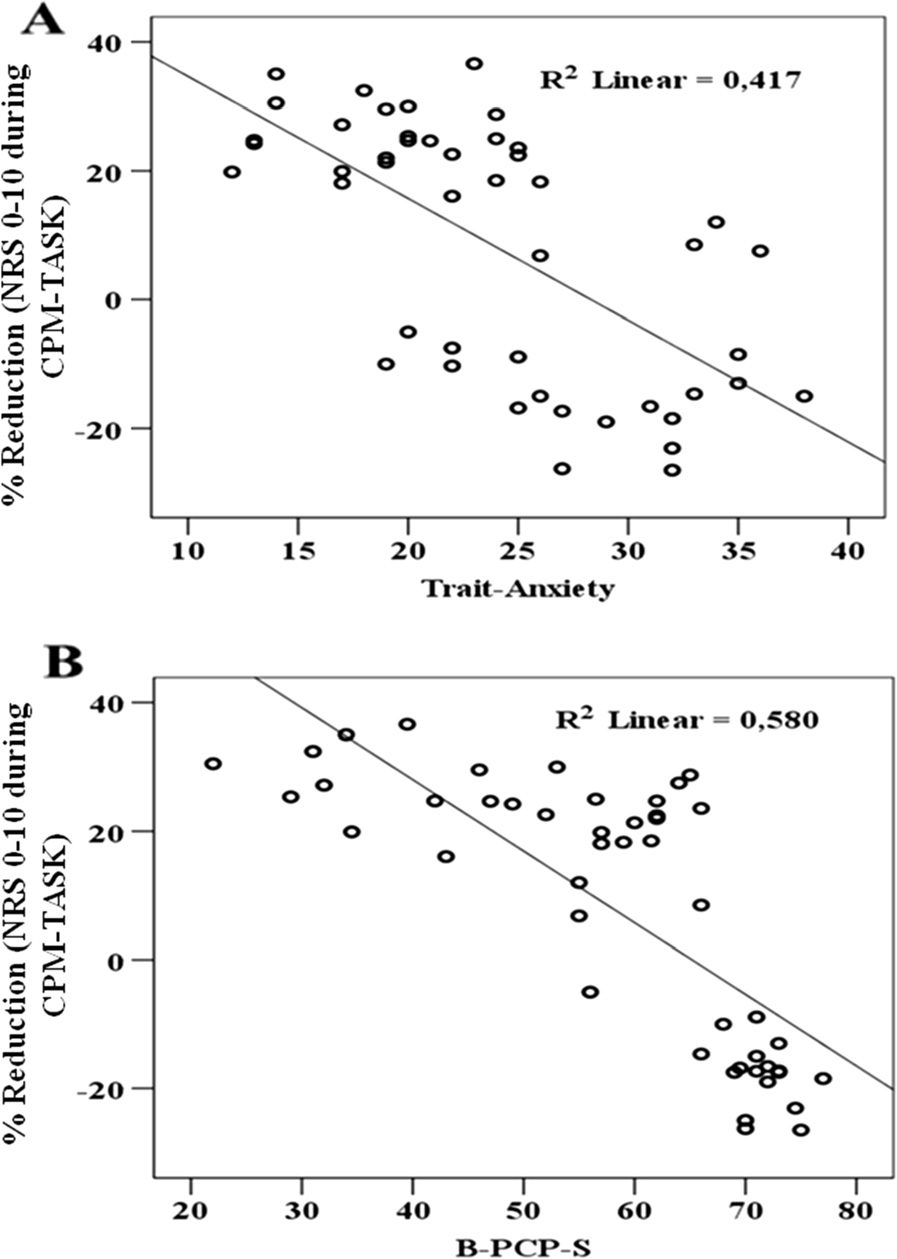
**Relationship between the percentage of pain reduction during the cold-pressor task (CPM-TASK on the numerical pain scale (NPS **_**(0-10)**_**) and the Disability related to pain (DRP) (A), and the trait-anxiety (B), (n = 47).**

The relationship between the percentage in pain reduction in NPS with trait-anxiety and DRP is presented in Figure 4. It was observed an inverse correlation between NPS during CPM-TASK in both DRP and trait-anxiety. This association between CPM, trait-anxiety and DRP observed in this univariate analysis was confirmed using multiple linear regressions (Table 5). This multivariate analysis confirmed that there is a significant inverse correlation between NPS during CPM-TASK and DRP, whereas the trait-anxiety was marginally significant (P = 0.05, see Table 5). A significant interaction between trait-anxiety and DRP was detected, when adjusting for this interaction one unit increment on this product (trait-anxiety vs. DRP) was associated with 39% increment on the NRS (P = 0.02) (Table 5). That is, the efficiency of the corticospinal system was reduced for modulating the pain evoked by QST. Also, this model showed that analgesics use did not change the relationship between anxiety levels and corticospinal excitability (P > 0.05).

**Table 5:** Relationship between CPM, trait-anxiety level and pain on the B-PCP:S (n = 47)

**Source**	**Type III sum of squares**	***df***	**Mean square error**	**F**	***P***
Corrected model	24327.85	4	26219.170	2.05	0.10
**Dependent variable: Percentage of change in the pain score on numerical pain scale during CPM-TASK**^**a**^					
**Parameter**	**β**	**SEM**	***T***	***P***	***Partial eta squared***
Intercept	458.80	221.72	2.07	0.04	0.02
B-PCP:S^b^	−10.56	3.82	−2.76	0.01	0.15
Trait-anxiety	−21.55	10.72	−2.01	0.05	0.09
B-PCP:S* Trait-anxiety	0.39	0.17	2.38	0.02	0.12
Analgesic dose used weekly^c^	−0.03	0.05	−0.07	0.94	0.00

^*a*^*Conditional pain modulation (CPM).*

^*b*^*Profile of Chronic Pain: Screen for a Brazilian population (B-PCP:S).*

^*b*^*Number of days analgesics were used per week in the last three months (< 4 times/≥ 4 times).*

## Discussion

This study suggests that there is a relationship between motor cortex excitability and trait-anxiety. We found a positive correlation between trait-anxiety, the DRP and the ICF. Trait-anxiety was inversely correlated with the CSP. Additionally, we observed that when high trait-anxiety concurs with DRP, the effect of the heterotopic noxious stimulus on the pain induced during the QST was reduced (Table 5). High trait-anxiety is positively correlated with increased cortical excitability and decreased intracortical inhibition as assessed by ICF and CSP (Table 4).

According to previous reports, the inhibitory deficits mediated by GABAergic receptors associated with manifestations of anxious behavior are coherent with our findings [6,34,35]. A marked increase in ICF has been demonstrated in drug-naive subjects suffering post-traumatic stress disorder (PTSD) [36], as well as a short CSP in patients with obsessive-compulsive disorder [7]. Although the underlying mechanism is not clear, several neurobiological processes may explain these findings, as follows. The ICF originates from excitatory postsynaptic potentials (PSPs) mainly mediated by glutamatargic NMDA receptors [37]. Their latency is about 10 ms, consistent with the time course of intracortical facilitation [38]. Pharmacological studies support such observation, as NMDA receptor antagonists (i.e. dextromethorphan) decrease ICF [38]. Likewise, GABA-A agonists like benzodiazepines (e.g. Lorazepam) and barbiturates decrease the SICF. This supports the hypothesis that the first pulse elicits the GABA-A receptor-mediated short latency inhibitory postsynaptic potential (PSPs) in corticospinal and/or first order excitatory interneurons that inhibits the facilitatory interactions with the second pulse [39]. On the other hand, GABA-B agonists (e.g. Baclofen) increase ICF [40]. It has also been suggested that ICF is not exclusively mediated by excitatory interneurons, but rather by a balance between inhibition and excitation [38].

Finally, the duration of the cortical inhibition assessed by CSP is consistent with intracellular measurements of the inhibitory PSPs from the stimulation of the GABA-A receptor [41,42]. Also, a short-lasting CSP shortening after diazepam injection has been reported [43]. It has been hypothesized that, just after diazepam injection, a GABA-A mediated inhibition could transiently reduce facilitatory thalamo-cortical influences on inhibitory interneurons of the motor cortex [43]. Overall, the changes in the balance between cortical inhibitory and facilitation observed in the present study may be explained by impairments in neurotransmission mediated by GABA-B and NMDA receptors [44].

We found that trait-anxiety explained about 36% of the variance of the MEP amplitude induced by QST evoked pain. Patients with high trait-anxiety might have less corticospinal modulation of the pain response during QST, which also made them present higher motor cortex excitability. Furthermore, it is unlikely that time or other non-specific effects of the QST intervention might have influenced the cortical excitability parameters, because healthy controls MEPs remained unchanged after the QST intervention. This finding is in agreement with previous reports that showed a short latency during a stimulus on peripheral sensory nerves induced by QST in anxious subjects [45]. Previous reports had consistently described increments on MEP after painful experiences [46-48] and under experimental pain [48-51]. To the best of our knowledge, the present study is the first assessment of the association between anxiety and MEP changes in patients with chronic pain submitted to standardized nociceptive stimuli. Thereby this finding suggests that the inhibitory capacity of the corticospinal modulator system is reduced. In fact, this effect in chronic pain patients underscores dysfunctional cortical processing in these subjects, because the motor reactions to pain in healthy controls result in the suppression of MEP amplitude from all distal hand muscles [48-51]. Another potential explanation to be considered is the potential protective effect of emotional amplification on pain consistent with the Gray–McNaughton theory, which proposes that during anxiety the hippocampal formation increases the valence of aversive events to prime behavioral responses adaptive to the worst possible outcome. It has been described that the hippocampal formation is responsible for increased pain by amplifying signals to the neural representation of the painful stimulus [52]. In fact this response may change overall processing of pain on the inhibitory system as compared to experimental pain (or acute pain) in healthy subjects. Overall, our observations help to interpret the electrophysiological evidence for the relationship between psychological symptoms and pain modulation.

Furthermore, it was also observed that the interaction between pain and anxiety reduced the CPM during the QST evoked pain. Considering that the CPM evaluates the function of the corticospinal system, a lower pain threshold was observed when the high trait-anxiety was concurrently with high DRP, this suggest that the function of the descending modulatory system was reduced. Such a finding is in agreement with previous reports in other chronic pain conditions, such as temporomandibular disorder [53]; fibromyalgia [54,55]; tension-type headache [56]; migraine [57] and also in healthy subjects with significant pain history [58]. Overall, these findings indicate that the impact of trait-anxiety on pain is linked with the central sensitization of nociceptive neurons, which contribute to the worsening of chronic pain symptoms. Nevertheless, when assessing the association between trait-anxiety and Diffuse Noxious Inhibitory Control (DNIC) induced by the immersion of one’s hand at 12°C [58,59]. Edwards *et al* did not find an association between the extent of endogenous analgesia (tested by DNIC) and psychological parameters, including the profile of mood states, locus of control, level of vigilance, and stress. Such divergence between our findings and those of Edwards *et al.*[58,59] could be explained by differences in the samples (such as the inclusion of healthy male subjects), the intensity of the stimuli used (12°C vs. 0°C) and type of assessment conducted.

We observed not only evidence of increased central sensitization in chronic pain associated with anxiety but also a negative impact of trait-anxiety on the endogenous modulatory system in patients with long-term pain associated with higher levels of disability. Thus, a potential mechanism to explain such effects may be related to a change in the neural response from amygdala. Healthy subjects’ pain modulation is attributed to endogenous opioid activity activated by the amygdala [60]. The amygdala is directly connected to brainstem structures responsible for the descending modulation, which is at the same time regulated by endogenous opioid activity [60]. Thus, in patients with chronic pain, although greater activation of the amygdale might occur, a reduced opioid activity is expected because of the central sensitization induced by the chronic condition, leading to an attenuated endogenous analgesic response [61]. Such hypothesized explanatory mechanisms for our observations presented here might make us consider the endogenous modulation system as a potential target for specific approaches in chronic pain control in the context of higher anxiety levels.

This study has some limitations. First, the use of TMS for neurophysiological assessments involved the evaluation of neurotransmitter system activity in an indirect manner, and it has been shown to have relatively low specificity. However, TMS provides a useful tool for neurophysiological assessment because it induces activity and evaluates a subject’s response. Second, we included only female subjects, although an enhanced pain response in females has been attributed to physiological and psychological variables, including mechanisms of endogenous inhibition, the capability to endure pain, genetic factors, pain expectation and personality traits [62,63]. In this context, the gender may be an important confounding factor because the amygdala is more prone to activation upon negative emotional responses (i.e., stress, fear, and anxiety) in females [64]. Another factor to consider is the hormonal variation throughout the menstrual cycle. Although this factor is a possible confounder, the effect of estrogen levels on the MEP after QST evoked pain can be minimized because each patient served as her own control. Third, another limitation to be considered is the way our team evaluated the use of analgesics because it is always possible to face a memory bias when inquiring patients about past events. It is possible that those experiencing more pain, or those with higher anxiety traits, would report a higher requirement of analgesics than those who were less anxious or experiencing less pain. Fourth, although we recruited healthy volunteers to assess the potential effect of time during the experiment, it is worth noting that our control sample was younger on average. Nevertheless, age only showed an effect on the motor threshold in the regression models, but not in the other cortical excitability parameters. Thus, it is unlikely that controls’ age would modify the present findings. Finally, it would be worthwhile to design similar studies to investigate anxiety’s effect on other chronic pain syndromes to confirm the findings revealed in the present study because our conclusions can only be claimed as valid in similar severe chronic MPS samples. Fifth, chronic pain is strongly associated with anxiety symptoms; this potential confounding factor cannot be fully controlled, as it is part of chronic pain syndromes [65,66]. It is important to emphasize that in the single-subject design the patients serve as own control. This design that is sensitive to individual organism’s differences permits to assess causal relations between the independent and dependent variables [67,68]. Whereas it reduces the potential of comparing with healthy subjects, it is an ideal strategy to validate results because in real life a scenario is complex to find controls that match with the profile of the chronic pain patients.

## Conclusions

In conclusion, we showed that when the chronic MPS concurs with high trait-anxiety and higher DRP pain, an imbalance occurs between excitatory and inhibitory impulses in the descending systems to the dorsal horn (as indexed by ICF, CSP and MEP change upon pain); thus providing further support to the effects of the emotional system on central sensitization and also to the potential mechanisms of decreased pain modulation in chronic pain.

## Abbreviations

MEP: Motor-evoked potentials; SICI: Short intracortical inhibition; OCD: Obsessive-compulsive disorder; MPS: Myofascial pain syndrome; MTrPs: Myofascial trigger points; CPM: Conditioned pain modulation; TMS: Transcranial magnetic stimulation; QST: Quantitative sensory testing; PTSD: Post-traumatic stress disorder; PSP: Postsynaptic potential; HCPA: Hospital de Clínicas de Porto Alegre; DN4: Neuropathic Pain Diagnostic Questionnaire; B-PCS: Brazilian Portuguese Catastrophizing Scale; DRP: Disability related to pain; B-PCP:S: Screen for a Brazilian Population; STAI: State-Trait Anxiety Inventory; VAS: Visual analogue scale; HPT: Heat pain threshold; SDM: Standardizing difference mean; MT: Resting motor threshold; CSP: Cortical silent period; ICF: Intracortical facilitation.

## Competing interests

The authors report no conflicts of interest with the collection or reporting of the results presented in this manuscript.

## Authors’ contributions

AD, LD, AB, JRR, GL, LM participated in the sequence alignment. JADS, WC participated in the design of the study and performed the statistical analysis. WC, FF, JADS, IT conceived the study, and participated in its design and coordination and helped to draft the manuscript. All authors read and approved the final manuscript.
